# Programming of One- and Two-Step Stress Recovery in a Poly(ester urethane)

**DOI:** 10.3390/polym9030098

**Published:** 2017-03-10

**Authors:** Nikolaus Mirtschin, Thorsten Pretsch

**Affiliations:** 1 BAM Federal Institute for Materials Research and Testing, Division 6.5, Polymers in Life Science and Nanotechnology, Unter den Eichen 87, 12205 Berlin, Germany; n.mirtschin@posteo.de; 2 Fraunhofer Institute for Applied Polymer Research IAP, Synthesis and Polymer Technology, Potsdam-Golm, Geiselbergstraße 69, 14476 Potsdam-Golm, Germany

**Keywords:** stimuli-sensitive polymer, stress-memory polymer, temperature-memory polymer, shape-memory polymer, poly(ester urethane), programming, thermoresponsiveness, stress recovery, stress memory

## Abstract

This work demonstrates that phase-segregated poly(ester urethane) (PEU) with switching segments of crystallizable poly(1,4-butylene adipate) (PBA) can be programmed to generate two separate stress recovery events upon heating under constant strain conditions. For programming, two elongations are applied at different temperatures, followed by unloading and cooling. During the adjacent heating, two-step stress recovery is triggered. The results indicate that the magnitude of the stress recovery signals corresponds to the recovery of the two deformation stresses in reverse order. As demonstrated by further experiments, twofold stress recovery can be detected as long as the elongation at higher temperature exceeds the strain level of the deformation at lower temperature. Another finding includes that varying the lower deformation temperature enables a control over the stress recovery temperature and thus the implementation of so-called “temperature-memory effects”. Moreover, exerting only one elongation during programming enables a heating-initiated one-step stress recovery close to the deformation temperature. Based on these findings, such polymers may offer new technological opportunities in the fields of active assembly when used as fastening elements and in functional clothing when utilized for compression stockings.

## 1. Introduction

Some materials can be programmed to fix a temporary shape and either recover shape or build up mechanical stress in response to heating. This behavior is characteristic for shape-memory materials [1], like shape-memory alloys [2,3,4] and shape-memory polymers (SMPs) [5,6,7,8,9]. Regarding the latter, the ease of manufacturing, low density, wide range of switching temperatures and the ability to perform complex movements on demand offers a variety of design opportunities. Therefore, SMPs have attracted significant attention in actuation [10,11,12], deployable structures [13,14,15,16,17], temperature sensing devices [18,19,20,21,22,23] and switchable information carriers [24,25,26,27,28]. 

Shape-memory polymers like phase-segregated polyurethanes are able to fix strong elongations and recover strains of several hundred percent. The beneficial thermomechanical behavior derives from network structure and the separation of immiscible hard and soft segments at room temperature [29,30,31]. Basically, the shape-memory effect (SME) can be programmed at temperatures around the phase transition temperature *T*_trans_ of the soft segment. Therefore, the polymer is heated from below to above *T*_trans_, elongated and cooled in the imposed shape below *T*_trans_. The fixation of the new shape is achieved by crystallization or vitrification of the soft segment, which stabilizes the entropically unfavorable state. When triggering the SME by heating, the soft segment serves as switching segment and entropy elasticity drives shape recovery. The polymer’s response to heating is commonly investigated under stress-free recovery conditions [29,32,33,34] or under constant strain recovery conditions [35,36,37,38,39]. The most relevant material parameters obtained from thermomechanical measurements are the strain fixity ratio, the strain recovery ratio and the maximum recovery stress. To date, high strain recovery ratios exceeding values of 85% are not unusual for thermoplastic SMPs [22,34,40], same as for thermoset SMPs [33,41,42]. However, the design of stress recovery behavior is in fact challenging. Concepts aiming at an increase in recovery stress include the incorporation of reinforcing nanofillers [35,37,43,44,45,46] and the adjustment of programming conditions. In terms of the latter, a raise in maximum strain [37,47,48], an extension in temperature holding time after deformation [49] and the selection of a deformation temperature below *T*_trans_ [30,50,51] turned useful. Apart from that, control over stress recovery behavior in the sense of the implementation of the temperature-memory effect (TME) has been documented for amorphous polymers [35,52] and for semicrystalline polymers [22,53]. As is most characteristic for such temperature-memory polymers, a substantial mechanical response is generated when they are heated above that temperature, at which a previous deformation has been carried out. 

For a couple of years, the knowledge about the programming of shape-memory polymers has been drastically extended. As a result, not only transitions from one shape into a second, but also from a second shape into a third one could be realized efficiently [54,55,56,57]. For switching processes, which are characterized by two or even more consecutive shape changes, the term “multiple-SME” had been coined [58]. A heating-initiated two-step strain release, for instance, could be witnessed after deforming a physically cross-linked poly(ester urethane) (PEU) at a temperature where the switching segment was first amorphous, before a second loading was applied in the semicrystalline state at a lower temperature, followed by further cooling, which led to the vitrification of the switching segment [59]. Later on, this triple-shape concept could be extended to chemically cross-linked PEU [60]. Alternatively, materials characterized by either a broad glass transition [61,62] or a wide melting/crystallization transition [63,64] have been used to program and trigger the SME. Intriguingly, the multiple-SME was not verified so far under constant strain recovery conditions. The only indication of coexisting stress signals was given by Grillard et al. for fibers made of polyamide 12 loaded with multiwall carbon nanotubes [52]. However, in this case the stress recovery behavior was characterized by a stress signal, which covered a broad temperature range of more than 100 °C.

To trigger two consecutive recovery events for the first time under constant strain conditions, the stress recovery behavior of a phase-segregated PEU containing crystallizable segments of poly(1,4-butylene adipate) (PBA) was investigated. For this purpose, a recently introduced programming route for temperature-memory effects [22] was extended by a second programming step. Against this background, it will be demonstrated in how far the variation of maximum strain is helpful to control stress recovery behavior and that the TME can be implemented in the stress recovery paths at lower temperatures.

## 2. Materials and Methods

### 2.1. Materials

The herein-investigated material was Desmopan 2795A SMP, which is a physically cross-linked poly(ester urethane) (PEU) from Covestro AG, Leverkusen, Germany. Samples were received as injection-molded plaques with a thickness of 2 mm. The hard segment was composed of 4,4′-methylenediphenyl diisocyanate (MDI) and of 1,4-butanediol (BD) used as chain extender. The soft segment was composed of poly(1,4-butylene adipate) (PBA), which was characterized by a molecular weight of 3500 g·mol^−1^. Detailed information regarding the thermal, mechanical and structural properties of the PEU was previously reported [22,49].

### 2.2. Characterization Methods

The phase transition behavior of the PEU was studied by differential scanning calorimetry (DSC) using an EXSTAR DSC7020 from Seiko Instruments Inc., Chiba, Japan. The sample weight was approximately 5 mg. In every measurement, the sample was first cooled to −90 °C, before it was heated to 90 °C. Cooling and heating were carried out with a rate of 10 °C·min^−1^. 

Thermomechanical measurements were conducted with an electromechanical testing system (Z005 from Zwick GmbH & Co. KG, Ulm, Germany), which was equipped with a thermochamber (Zwick GmbH & Co. KG, Ulm, Germany) and a temperature controller (Eurotherm 2261e, Eurotherm Deutschland GmbH, Limburg, Germany). Test procedures were designed with the software testXpert^®^ II (V 3.31, Zwick GmbH & Co. KG, Ulm, Germany). For specimen preparation, type 5B tensile bars (DIN EN ISO 527-2:1996) were punched out of the PEU plaques. Prior usage, specimens were annealed for 10 min at 60 °C and stored for at least one week at 23 °C and at an air humidity of 50%. Before starting a measurement, a tensile bar was clamped with a gauge length of 10 mm into the pneumatic grips of the electromechanical testing system. Typically, a clamping pressure of 5.2 bar was selected. During a measurement, changes in normal force were detected with a 100 N load cell. In parallel, the stress σ was determined by dividing the force through the initial cross-section of the specimen. Changes in strain ε were followed from crosshead displacement. Uniform heating and cooling rates of 3 °C·min^−1^ were used. After heating, cooling and deformation, a temperature holding step of 5 min was added.

In a first programming series, a PEU specimen was heated to the deformation temperature *T*_d,high_ = 40 °C, elongated with a strain rate of 3 × 10^2^% min^−1^ to a maximum strain ε_m,high_ of 400%, held at 40 °C for 5 min and unloaded with a rate of 1 × 10^3^% min^−1^. Subsequently, the specimen was cooled to *T*_d,low_ = 0, 10 or 20 °C and elongated with a strain rate of 3 × 10^2^% min^−1^ to ε_m,low_ = 225%, 275%, 325%, 375% or 425%. After elongation, the imposed strain was maintained for 5 min and the residual stress was removed with a rate of 1 × 10^3^% min^−1^. In another experiment, the first programming step was maintained, but the temperature holding time after the second deformation (*T*_d,low_ = 0 °C, ε_m,low_ = 225%) was extended from 5 min to 15 h, before unloading was carried out. In any case, programming was finalized by cooling to −20 °C. The fixed strain was determined after unloading at *T*_d,high_ and *T*_d,low_ and was termed “ε_u,high_” and “ε_u,low_”, respectively. 

The thermoresponsiveness of the PEU was adjacently studied under constant strain recovery conditions. Therefore, the specimen was heated from −20 to 80 °C with a rate of 3 °C·min^−1^. In those cases, in which the specimen responded with two separate stress recovery events, the stress recovery temperatures were determined by using a two-tangent intersection method. Therefore, tangent lines were drawn in the recovery curve of the stress-temperature test protocol and the temperatures corresponding to their intersection points at the beginning of the first and the second stress increase were defined as onset temperatures *T*_σ,low_ and *T*_σ,high_. Apart from that, maximum recovery stresses were quantified and denominated as “σ_max,low_” and “σ_max,high_” in accordance with the corresponding temperatures *T*_σ,max,low_ and *T*_σ,max,high_. In those cases, in which no local stress maximum could be detected, the inflection point between *T*_σ,low_ and *T*_σ,high_ was used to define the temperature *T*_σ,max,low_ and the associated stress σ_max,low_. 

In a different scenario, a one-step programming approach was followed. In this case, a PEU specimen was heated to 40 °C, elongated with a rate of 3 × 10^2^% min^−1^ to a maximum strain of 400%, held at 40 °C for 5 min and unloaded with a rate of 1 × 10^3^% min^−1^. Programming was finalized by cooling to −20 °C. Subsequently, the specimen was heated to 80 °C under constant strain recovery conditions, using a rate of 3 °C·min^−1^. 

## 3. Results and Discussion

**Figure 1:**
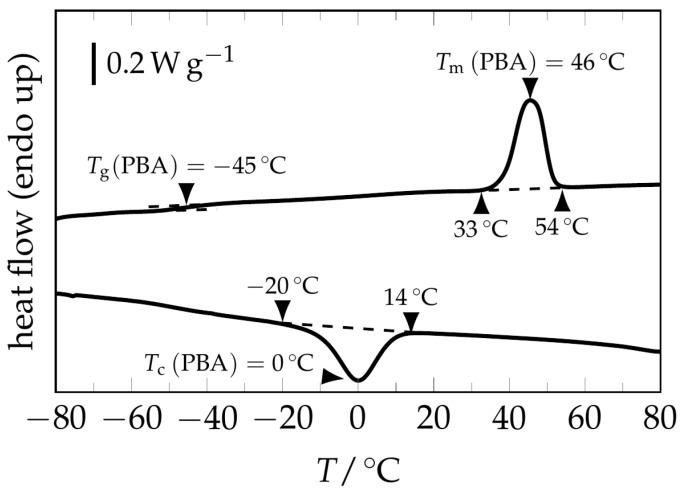
DSC thermogram of the studied poly(ester urethane) (PEU). The first heating scan (above) shows the glass transition and a signal associated with the melting of the poly(1,4-butylene adipate) (PBA) phase, while the ensuing cooling scan (below) exhibits a signal related to the crystallization of the PBA phase. For heating and cooling, rates of 10 °C·min^−1^ were selected.

The calorimetric properties of the poly(ester urethane) (PEU) were characterized by a melting transition between 33 and 54 °C, a crystallization transition spreading from 14 to −20 °C, and a glass transition at about −45 °C (Figure 1); all of these phase transitions could be assigned to poly(1,4-butylene adipate) (PBA), which later served as switching segment in the PEU [22].

**Figure 2:**
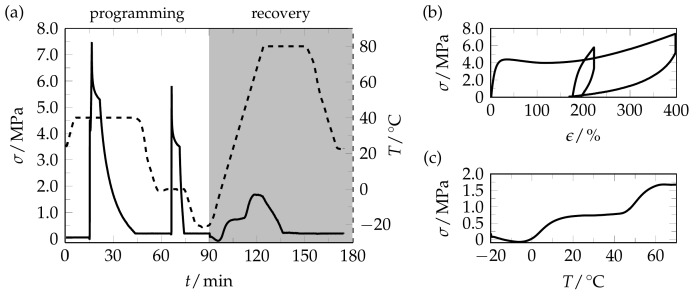
Twofold programming and stress recovery behavior of PEU. (**a**) Stress (**solid line**) and temperature (**dashed line**) vs. time protocols. Programming consisted of deformation and unloading both at 40 and 0 °C, followed by cooling to −20 °C. The stress recovery behavior was investigated during heating to 80 °C. Finally, the specimen was unloaded and cooled to 23 °C. Heating and cooling were accomplished with a rate of 3 °C·min^−1^. (**b**) Stress-strain relation when applying two-step programming, including an elongation to 400% at 40 °C and unloading, same as the application of an absolute maximum strain of 225% at 0 °C and unloading. The associated loading and unloading rates were 3 × 10^2^ and 1 × 10^3^% min^−1^, respectively. (**c**) Stress recovery behavior of twofold programmed PEU during heating from −20 to 70 °C.

Having these thermal properties in mind, two-step programming was applied at temperatures around the melting and crystallization transition of the PBA phase, and the resulting stress recovery behavior was investigated (Figure 2). 

As an essential part of programming, the semicrystalline PEU was twofold elongated, first to a strain ε_m,high_ of 400% at 40 °C, and then to a strain ε_m,low_ of 225% at 0 °C. The specimen behavior during programming and the adjacent recovery behavior during heating under constant strain conditions are given in Figure 2a. In contrast to earlier two-step programming approaches [52,59,61,65], unloading was carried out at the end of every deformation step. In the course of first deformation, a strong increase in stress after the yield point occurred as can be clearly seen in the associated stress-strain diagram (Figure 2b). This observation speaks for the occurrence of strain-induced PBA crystallization [10]. A similar behavior had been witnessed by Tobushi et al. for polyurethanes with not specified polyester polyol segments [66]. After the adjacent unloading, a fixed strain ε_u,high_ of 176% was determined, suggesting that the freshly formed PBA crystals blocked the elastic recovering into the original shape. An additional strain-hardening seemed to occur during the second deformation. As a result of the ensuing unloading, the fixed strain ε_u,low_ could even be raised to 198%. Programming was finalized by cooling to −20 °C, where the specimen was kept for 5 min. The thermoresponsiveness was adjacently investigated (Figure 2c). In the early phase of heating, the polymer built up a slight amount of compressive stress. This behavior originated from thermal expansion of the specimen as earlier witnessed for the same material [49]. Further heating initiated stress recovery, which started at −1 °C and thus close to the second deformation temperature. At approximately 27 °C, a stress plateau emerged at about 0.7 MPa. Heating beyond 27 °C resulted in a second stress response, which started at 45 °C close to the first deformation temperature and culminated in an overall recovery stress of 1.7 MPa, which was reached at 64 °C. This way, a two-step stress release could be witnessed starting with a lower stress, as expected from the lower maximum stress applied during the second programming step, followed by a higher stress in line with the higher maximum stress applied during the first programming step. Although none of the applied stresses could be fully recovered, the order of stress recovery was taken as hint that the PEU exhibited stress-memory properties. However, it should be noted that the PEU specimen memorized only a small part of the stresses exerted during programming. Regarding the first recovery event, the maximum recovery stress reached 13% of the corresponding stress applied during deformation at *T*_d,low_. In the case of the second recovery event, 23% of the maximum stress exerted during deformation at *T*_d,high_ could be regained.

**Figure 3:**
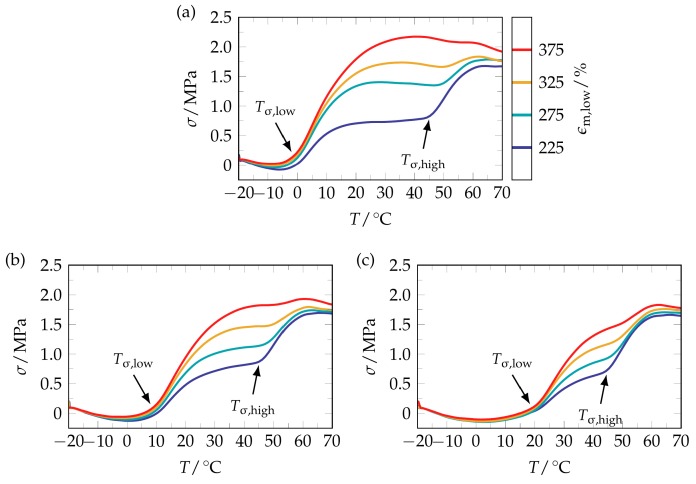
Stress recovery behavior of twofold programmed PEU when varying the deformation conditions in the second programming step (not shown here). In detail, first programming included the application of a maximum strain of 400% at *T*_d,high_ = 40 °C, unloading and cooling to the second deformation temperature, while second programming consisted of different elongations ε_m,low_ (color coded) at 0 °C (**a**), 10 °C (**b**) and 20 °C (**c**). In all cases, the stress recovery temperatures *T*_σ,low_ and *T*_σ,high_ are drawn in.

To demonstrate that the twofold stress recovery behavior of the PEU can be controlled by the deformation conditions, the first programming step was left unchanged and the second programming step was modified. Against this background, deformation temperatures of 0, 10 and 20 °C and maximum strains of 225%, 275%, 325% and 375% were investigated. The results are provided in Figure 3 and in Table 1. 

**Figure 4:**
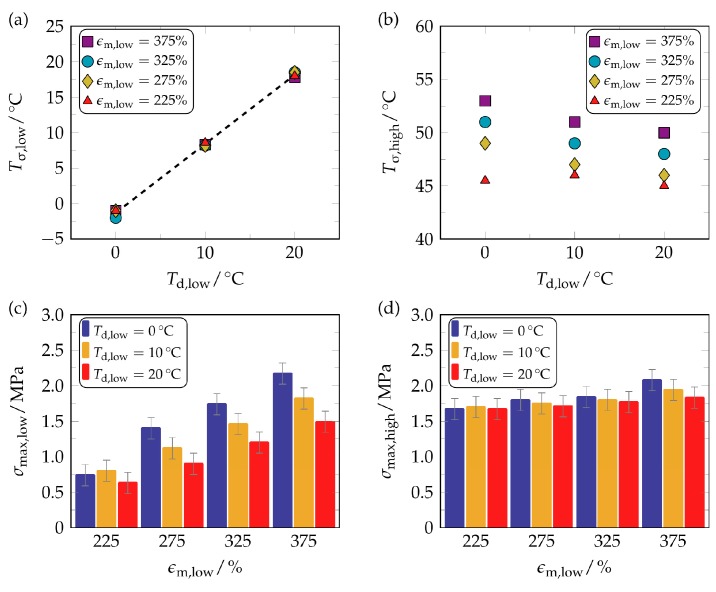
Evaluation of the two-step stress-recovery behavior of PEU: Influence of twofold programming when varying the lower deformation temperature *T*_d,low_ and the maximum strain applied ε_m,low_. Relation between *T*_d,low_ and the stress recovery temperature *T*_σ,low_ ((**a**), with best fit line) and between *T*_d,low_ and the stress recovery temperature *T*_σ,high_ (**b**). Impact of ε_m,low_ upon both maximum recovery stress σ_max,low_ (**c**) and maximum recovery stress σ_max,high_ (**d**). The errors in (**c**,**d**) were estimated from repeated measurements.

Most obviously, a twofold stress increase can be seen in all measurement protocols (Figure 3). Beyond that, it is noteworthy that those temperatures, at which the release of stress started, were close to the lower deformation temperatures. This way, the polymer exhibited time and again a “stress-related” temperature-memory effect (TME). For a more quantitative evaluation of the stress recovery behavior, the recovery temperatures and the recovery stresses were determined and processed into graphic form (Figure 4).

Figure 4a shows that the stress recovery temperature *T*_σ,low_ increased linearly with the lower deformation temperature *T*_d,low_, which impressively demonstrates that *T*_σ,low_ was independent from the strain applied during the second loading. As stated above, such a relationship satisfies the criterion of temperature-memory behavior [35]. On the other hand, the second stress recovery temperature *T*_σ,high_ consistently exceeded the higher deformation temperature *T*_d,high_ of 40 °C and even increased steadily with the maximum strain applied, no matter which *T*_d,low_ had been selected (Figure 4b). Since recovery stresses generally increased at stronger deformations (Figure 4c), the material stiffness must have increased as a result of the second loading. This probably affected the thermal stability of the PBA crystallites, leading to a shift in PBA melting temperature toward higher values, and thus to an increase in the associated stress recovery temperature *T*_σ,high_ (Figure 4b). Apart from that, the maximum strain applied during the low temperature deformation was found to be crucial for the first stress recovery event. For almost all stresses exerted at low temperature (Figure 4c), the associated recovery stresses decreased when raising the lower deformation temperature. This behavior was expected, since lower external loads were necessary in the preceding programming step, so that the amount of fixed strain after the second unloading ε_u,low_ was also reduced (Table 2).

Basically, a decreasing stiffness could be expected when specimens were deformed at higher temperatures, as such behavior applies to most polymers [67]. Unlike the first stress recovery, which could be controlled by the maximum strain exerted at lower temperature, the applied maximum strain did almost not affect the second stress recovery; in particular, its influence upon σ_max,high_ seemed to be negligible (Figure 4d). For this reason, it can be concluded that the second stress recovery must have been strongly influenced by the initial programming step.

**Figure 5:**
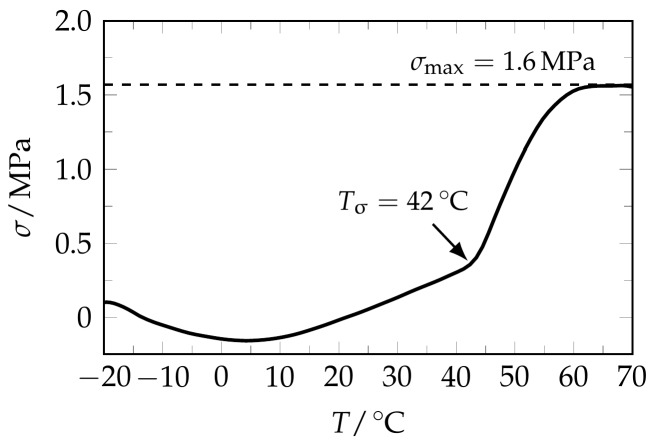
Stress recovery behavior of one-step programmed PEU. Programming included an elongation to a maximum strain of 400% at *T*_d_ = 40 °C, unloading and cooling to −20 °C. The stress recovery temperature *T*_σ_ is marked. It was determined from the two-tangent intersection method. The maximum recovery stress σ_max_ is also drawn in.

In order to gain a deeper understanding for the overall stress recovery behavior, one further thermo-mechanical measurement was carried out. This time, only a single programming step was applied by elongating a PEU specimen to a maximum strain of 400%, unloading it at 40 °C and cooling it to −20 °C. Subsequently, the stress response during heating to 70 °C was investigated. The associated stress-temperature diagram (Figure 5) shows that the PEU started to significantly build up stress at 42 °C, which is close to *T*_d_, and therefore proves that a stress-related temperature-memory effect could be programmed. 

Upon further heating, a maximum recovery stress of 1.6 MPa was reached at 65 °C. In comparison with the stress recovery of the twofold programmed specimen presented in Figure 2c, a one-step stress release could be witnessed here. In both cases, programming presumably supported the occurrence of additional crystallization as facilitated by strong molecular chain alignment upon loading to high maximum strains. It is known from programming single TMEs in the same material that the crystalline PBA phase, which is thermally stable at temperatures above the deformation temperature, fixes the polymer chains in their elongated states [22]. Apparently, the same fundamentals apply to twofold programmed PEU: The melting of PBA crystals that were thermally stable at *T* ≥ *T*_d,low_ affected the overall stress recovery behavior, including σ_max,low_ and σ_max,high_. 

**Figure 6:**
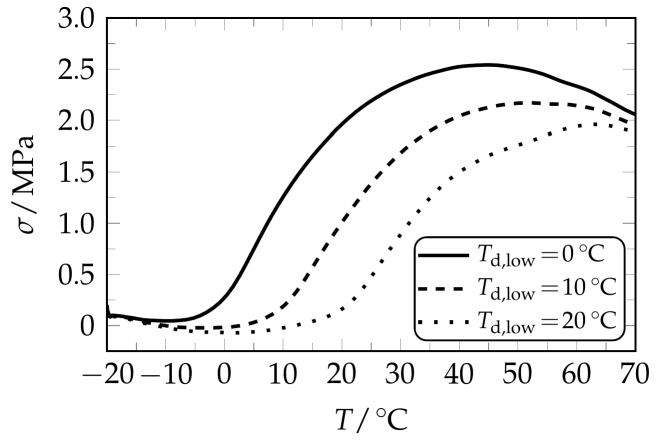
Stress recovery behavior of twofold programmed PEU. The second elongation was larger than the first one. Stress recovery is shown for those specimens, which were elongated to 400% at 40 °C and to an absolute strain of 425% at 0 °C (**solid line**), 10 °C (**dashed line**) and 20 °C (**dotted line**).

Another experimental series was designed to find out in which way the thermoresponsiveness of the PEU can be influenced when applying a stronger deformation in the second programming step. Therefore, specimens were elongated to a strain of 400% at 40 °C before an absolute strain of 425% was applied at 0, 10 and 20 °C, respectively. The resulting stress-temperature responses are provided in Figure 6. 

It can be clearly seen that the stress responses were no longer characterized by two individual stress maxima, but by one broad stress recovery signal, which in every single measurement emerged close to *T*_d,low_ (Table 3). 

Due to the high maximum strain applied in the second programming step, a strong alignment of polymer chains must have occurred. This could have supported the formation of PBA crystals whose melting temperature was above *T*_d,low_ or corresponded to it. As a matter of fact, the overall stress recovery behavior was composed of one stress recovery event dominated by the programming at *T*_d,low_, while no second individual stress signal associated with the programming at *T*_d,high_ could be witnessed. Similarly, the formation of a broad stress recovery signal was also observed by Grillard et al. for twofold deformed fully amorphous fibers of polyamide 12 loaded with carbon nanotubes [52]. Interestingly, the elongation at lower temperature was also selected to exceed the strain level of the deformation at higher temperature, so that no individual stress-recovery signal at *T*_d,high_ could be detected.

**Figure 7:**
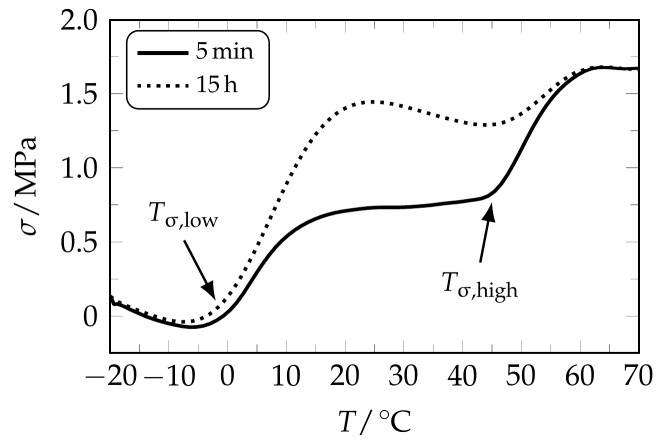
Stress recovery behavior of twofold programmed PEU when varying the temperature holding time after the second deformation. Twofold stress recovery is shown for those specimens, which were elongated to 400% at 40 °C and to 225% at 0 °C, whereupon the temperature holding time was extended from 5 min (**solid line**) to 15 h (**dotted line**).

Finally, it should be recognized that the recovery stress of polyurethanes can be designed by controlling the degree of crystallinity [50,68], as facilitated for instance by an extension of temperature holding time after deformation [49]. Therefore, a specimen was elongated to a strain of 225% at 0 °C and the time after elongation was exemplarily extended from 5 min to 15 h, before cooling was applied and the thermoresponsiveness was once more investigated (Figure 7). 

As a result, twofold stress recovery could be witnessed and the maximum recovery stress σ_max,low_ was doubled from 0.7 to 1.4 MPa. Remarkably, both the transition temperatures and the recovery stress at elevated temperature were not impaired by the modification of temperature holding time, which enhanced control over the twofold stress recovery behavior of semicrystalline PEU. 

## 4. Conclusions

Various two-step programming approaches were developed to investigate the stress-recovery behavior of semicrystalline polyurethane. Commonly, twofold stress recovery could be detected and was widely influenced by the deformation conditions selected at the lower deformation temperature. For instance, weaker elongation favored two stress signals, which could be fine-tuned by maximum strain and temperature holding time; in turn, stronger deformation resulted in a single and broad stress recovery signal upon continuous heating. The order of stress recoveries implied that the material exhibited stress-memory properties. Regardless of whether a one- or two-step programming approach was followed, an accurate setting of stress recovery temperatures was possible, which proved the temperature-memory behavior of the PEU. 

We anticipate that the witnessed control over stress recovery behavior is transferrable to other semicrystalline polymer systems. The results open new opportunities to design stress release through programming, which may broaden the field of vision for stress-memory polymers. Attractive applications may include active assembly and functional clothing.

## Figures and Tables

**Table 1:** Influence of the deformation conditions selected for the second programming step on the two-step stress recovery behavior of PEU. The deformation temperature *T*_d,low_ and the maximum strain at low temperature elongation ε_m,low_ were varied. The first programming step consisted of an elongation to 400% at 40 °C, unloading and cooling to the second deformation temperature *T*_d,low_. Errors were estimated from repeated measurements.

*T*_d,low_	ε_m,low_	*T*_σ,low_	*T*_σ,max,low_	σ_max,low_	*T*_σ,high_	*T*_σ,max,high_	σ_max,high_
(°C)	(%)	(°C)	(°C)	(MPa)	(°C)	(°C)	(MPa)
0	225	−1 ± 2	27 ± 2	0.7 ± 0.2	45 ± 2	64 ± 2	1.7 ± 0.2
0	275	−1 ± 2	28 ± 2	1.4 ± 0.2	49 ± 2	65 ± 2	1.8 ± 0.2
0	325	−2 ± 2	36 ± 2	1.7 ± 0.2	51 ± 2	62 ± 2	1.8 ± 0.2
0	375	−1 ± 2	40 ± 2	2.2 ± 0.2	53 ± 2	59 ± 2	2.1 ± 0.2
10	225	9 ± 2	39 ± 2	0.8 ± 0.2	46 ± 2	66 ± 2	1.7 ± 0.2
10	275	8 ± 2	41 ± 2	1.1 ± 0.2	47 ± 2	63 ± 2	1.8 ± 0.2
10	325	8 ± 2	45 ± 2	1.5 ± 0.2	49 ± 2	62 ± 2	1.8 ± 0.2
10	375	8 ± 2	47 ± 2	1.8 ± 0.2	51 ± 2	60 ± 2	1.9 ± 0.2
20	225	18 ± 2	40 ± 2	0.6 ± 0.2	45 ± 2	66 ± 2	1.7 ± 0.2
20	275	19 ± 2	42 ±2	0.9 ± 0.2	46 ± 2	65 ± 2	1.7 ± 0.2
20	325	19 ± 2	44 ± 2	1.2 ± 0.2	48 ± 2	64 ± 2	1.8 ± 0.2
20	375	18 ± 2	46 ± 2	1.5 ± 0.2	50 ± 2	62 ± 2	1.8 ± 0.2

**Table 2:** Influence of the deformation conditions selected for the second programming of PEU on the deformation stress σ_m,low_ and on the amount of fixed strain ε_u,low_. The deformation temperature *T*_d,low_ and the maximum strain at low temperature elongation ε_m,low_ were varied. The first programming step consisted of an elongation to 400% at 40 °C, unloading and cooling to the second deformation temperature *T*_d,low_. Errors were estimated from repeated measurements.

	*T*_d,low_ = 0 °C		*T*_d,low_ = 10 °C		*T*_d,low_ = 20 °C	
ε_m,low_ (%)	σ_m,low_ (MPa)	ε_u,low_ (%)	σ_m,low_ (MPa)	ε_u,low_ (%)	σ_m,low_ (MPa)	ε_u,low_ (%)
225	5.8 ± 0.2	198 ± 5	4.7 ± 0.2	187 ± 5	3.4 ± 0.2	186 ± 5
275	9.3 ± 0.2	215 ± 5	6.8 ± 0.2	212 ± 5	5.4 ± 0.2	202 ± 5
325	11.8 ± 0.2	245 ± 5	9.3 ± 0.2	235 ± 5	7.6 ± 0.2	221 ± 5
375	15.9 ± 0.2	269 ± 5	12.4 ± 0.2	256 ± 5	10.1 ± 0.2	241 ± 5

**Table 3:** Influence of the deformation temperature *T*_d,low_ on the deformation stress σ_m,low_, the amount of fixed strain ε_u,low_ and the stress recovery behavior of the PEU. Programming consisted of a first elongation to 400% at 40 °C, unloading, and an adjacent elongation to an absolute strain ε_m,low_ of 425% at *T*_d,low_, followed by unloading. Errors were estimated from repeated measurements.

*T*_d,low_ (°C)	σ_m,low_ (MPa)	ε_u,low_ (%)	*T*_σ,on_ (°C)	*T*_σ,max_ (°C)	σ_max_ (MPa)
0	19.5 ± 0.2	300 ± 5	−2 ± 2	45 ± 2	2.5 ± 0.2
10	15.4 ± 0.2	285 ± 5	8 ± 2	51 ± 2	2.2 ± 0.2
20	12.9 ± 0.2	266 ± 5	18 ± 2	63 ± 2	2.0 ± 0.2
